# The ImSURE phantoms: a digital dataset for radiomic software benchmarking and investigation

**DOI:** 10.1038/s41597-022-01715-6

**Published:** 2022-11-12

**Authors:** Andrea Bettinelli, Francesca Marturano, Anna Sarnelli, Alessandra Bertoldo, Marta Paiusco

**Affiliations:** 1 Medical Physics Department, Veneto Institute of Oncology – IOV IRCCS, Padova, Italy; 2 Department of Information Engineering, University of Padova, Padova, Italy; 3 IRCCS Istituto Romagnolo per lo Studio dei Tumori (IRST) “Dino Amadori”, Meldola, Italy; 4 Padova Neuroscience Centre (PNC), University of Padova, Padova, Italy

**Keywords:** Predictive markers, Tomography, Cancer

## Abstract

In radiology and oncology, radiomic models are increasingly employed to predict clinical outcomes, but their clinical deployment has been hampered by lack of standardisation. This hindrance has driven the international Image Biomarker Standardisation Initiative (IBSI) to define guidelines for image pre-processing, standardise the formulation and nomenclature of 169 radiomic features and share two benchmark digital phantoms for software calibration. However, to better assess the concordance of radiomic tools, more heterogeneous phantoms are needed. We created two digital phantoms, called ImSURE phantoms, having isotropic and anisotropic voxel size, respectively, and 90 regions of interest (ROIs) each. To use these phantoms, we designed a systematic feature extraction workflow including 919 different feature values (obtained from the 169 IBSI-standardised features considering all possible combinations of feature aggregation and intensity discretisation methods). The ImSURE phantoms will allow to assess the concordance of radiomic software depending on interpolation, discretisation and aggregation methods, as well as on ROI volume and shape. Eventually, we provide the feature values extracted from these phantoms using five open-source IBSI-compliant software.

## Abstract (summary)

**Table:** 

Measurement(s)	Radiomic Features
Technology Type(s)	Digital Phantoms and Radiomic Software Tools

## Background & Summary

Radiomics consists in the quantitative description of medical images through features that are able to uncover characteristics not perceivable with the naked eye. In oncology, radiomic features are investigated as possible biomarkers of tumour lesions and are included in the development of prognostic and predictive models^1^.

However, several challenges hamper the translation of radiomics into clinical practice, among which there is the lack of a standard method for radiomic features calculation. This deficiency hinders the reproducibility of radiomic studies and has recently called for a collective standardisation effort, which resulted in the International Image Biomarker Standardisation Initiative (IBSI)^2^ where twenty-five research groups reached a consensus for the calculation of 169 radiomic features. Two digital phantoms were employed for the task: the “*digital phantom*” (available at: https://github.com/theibsi/data_sets) and the “*radiomic phantom*”^3^, each one comprising one region of interest (ROI). The former was designed to be employed without any pre-processing ahead of feature calculation, and evaluates all possible aggregation methods. The latter was derived from a chest CT of a patient with non-small cell lung carcinoma and was designed to be used with five different pre-processing configurations, each one having either 2D or 3D feature aggregation approaches, presence or absence of interpolation and either fixed bin number (FBN) or fixed bin size (FBS) intensity discretisation method.

The initiative has entailed the standardisation of several open- and closed-source radiomic software and the publication of a number of studies aiming to assess feature reproducibility among radiomic tools^4–6^. Some of these works developed more heterogeneous digital phantoms by accounting for different ROI shapes and patterns^6^ to overcome the limited casuistry of the two IBSI phantoms. However, the majority of these works only considered a subset of the 169 IBSI-standardised features, typically those in common to the considered set of radiomic tools, and none of them systematically investigated software capability to set all combinations of pre-processing choices (e.g., interpolation, discretisation), nor their sensitivity to different ROI morphologies (e.g., shape and volume).

The aim of this work was to overcome this lack by proposing two heterogeneous digital phantoms and a systematic feature extraction that can become a reference to benchmark radiomic tools and can be used for an exhaustive investigation of software performances and agreement. The proposed phantoms were created in the context of the “*Italian multicentre Shared Understanding of Radiomic Extractors - ImSURE*” project^7^ and were designed to have both isotropic and anisotropic voxel size and to include 90 ROIs with different textures and morphologies. The phantoms are thought to be used in conjunction with a systematic workflow of feature extraction that allows a meticulous software investigation and comprises the calculation of all the 169 IBSI-standardised radiomic features and includes all the possible combinations of pre-processing and feature aggregation methods, for a total of 919 feature values computed for each ROI. For comparison, consider that IBSI phantoms had just one region of interest, hence they allowed a two-sample evaluation of each radiomic feature value (one for the digital and one for the radiomic phantom), which was essential to ease the standardisation process. Nevertheless, some implementation differences could go unrecognised, especially for the less complex IBSI digital phantom. Our 90-ROI phantoms reduce the likelihood of obtaining matching feature values by chance while also enabling the use of statistical tests to examine the effects of novel factors (such as shape and volume) on software agreement.

Reference feature values, which are shared together with the proposed ImSURE phantoms, were extracted using 5 radiomic tools (i.e. MIRP^8^, S-IBEX^9^, RaCaT^10^, SERA^11^ and Pyradiomics^12^) that were selected because of their wide usage in the radiomic field, and their flexibility in setting different parameter configurations. The ImSURE phantoms and the corresponding reference feature values could represent a useful tool to test and compare the agreement of a new radiomic software with those we proposed as a reference.

As further applications, the ImSURE phantoms could also be used to compare image filtering methods across radiomic software, which is of primary importance (https://theibsi.github.io/ibsi2/), as different implementations of the same filtering technique have repercussions on calculated feature values. In addition, one could also replicate the proposed feature extraction on the ImSURE phantoms with one of the five tools used in the present work, to gain expertise in setting program-specific parameters. Indeed, the task is not trivial as, depending on the software, the same configuration could be obtained with different settings. Eventually, the methodology presented in this paper could be reused to accurately position ROIs of any desired shape and volume inside a medical image to create new phantoms with different characteristics that could be useful for radiomics as well as for other fields.

## Methods

### Design of the ImSURE phantoms

#### Image retrieval, anonymisation and resample

A CT acquisition from skull base to mid-thigh of a patient randomly selected from a database of patients who signed informed consent was retrieved from the picture archiving and communication system (PACS) and anonymised using the Python library *DicomAnonymizer* (https://github.com/KitwareMedical/dicom-anonymizer). In addition to the standard anonymisation of DICOM fields offered by the routine, ‘*Instance Creation Date*’ and ‘*Instance Creation Time Attribute*’ fields were also overwritten to delete any reference to the date of the examination.

The original CT image had anisotropic voxels with a dimension of 0.98 × 0.98 × 3.00 mm and was used to create the ‘*ImSURE anisotropic phantom*’. An IBSI-compliant trilinear interpolation was then applied to the CT image to generate a second image with isotropic voxel dimension of 1.00 × 1.00 × 1.00 mm, which was used to build the ‘*ImSURE isotropic phantom*’.

#### Design of the ROIs

Each phantom was designed to contain 10 repetitions of 9 base morphologies, obtained as a combination of 3 shapes (i.e., cube, sphere and bean-like) and 3 volumes (i.e., small - *0.125 cm*^3^, medium - *1 cm*^3^, large - *8 cm*^*3*^), for a total of 90 ROIs. Cube and sphere shapes were chosen because of their simple and symmetric geometry, while the bean-like shape was introduced to mimic a morphology closer to that of a clinical ROI. The choice of the nine morphologies was made as part of the study design, and the necessity of having a sufficient number of ROIs (at least thirty) for statistical analyses when stratifying by shape or volume drove the number of repetitions. These decisions reflected a trade-off between the need to conduct a systematic study and to keep an overall manageable number of ROIs.

**Fig. 1:**
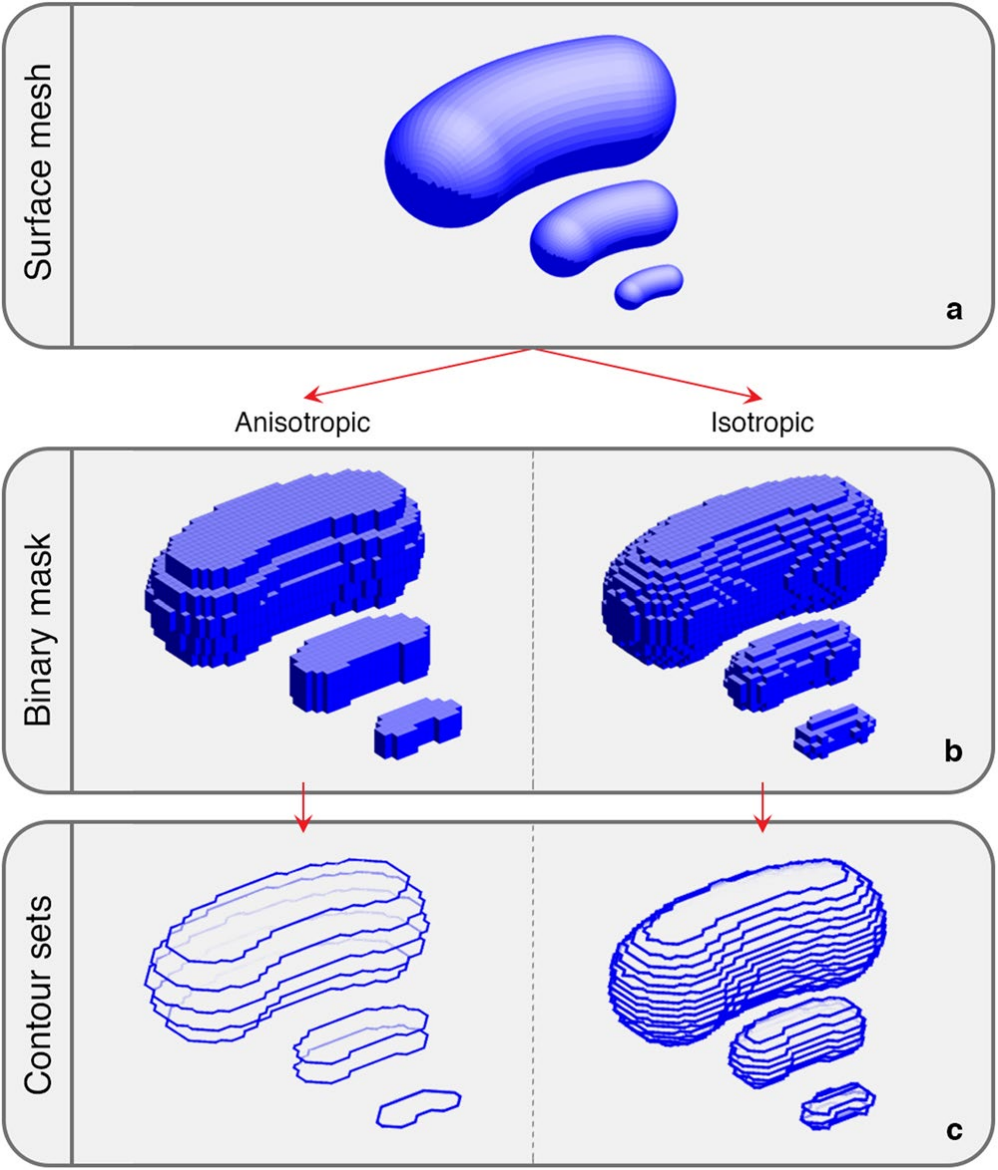
Steps of ROI creation for the bean-like shape. (**a**) Definition of bean-like 3D surface meshes; (**b**) Creation of binary masks for both the anisotropic and the isotropic ROI; (**c**) Definition of ROI contour sets.

ROIs were created with pinpoint accuracy down to the single-voxel level following three main steps: 1) design of surface models for each base morphology, 2) conversion of the surface models to binary masks and 3) definition of the ROI contour set (as required by the DICOM standard). The process is depicted in Fig. 1.

**Fig. 2:**
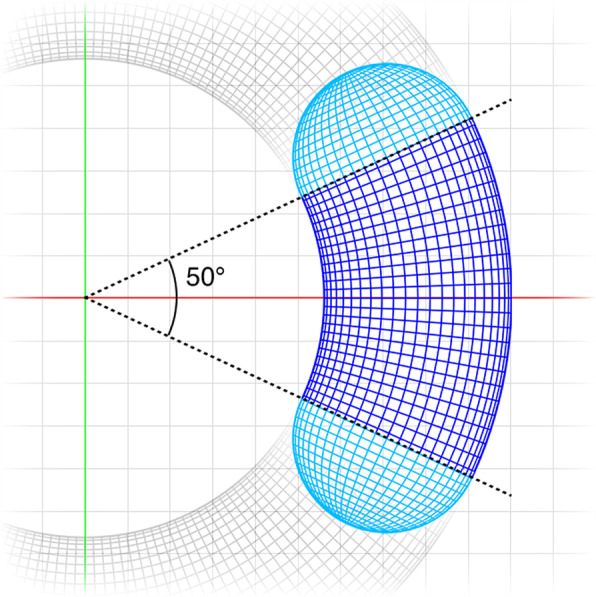
Orthographical top view of the bean surface mesh, obtained by capping a 50-degree torus section (blue) with two hemi-spheres (light blue).

Surface meshes were designed using Blender software^13^. The Blender built-in “primitive” meshes were used to realise the cubical and spherical ROI morphologies, while for the creation of the bean-like geometry, the extremities of a 50-degree torus section were capped with two half-spheres (Fig. 2). Each shape was then resized to obtain 3 predefined volumes (i.e. 0.125 cm^3^, 1 cm^3^, 8 cm^3^) and was exported as a separate*.stl* file.

**Fig. 3:**
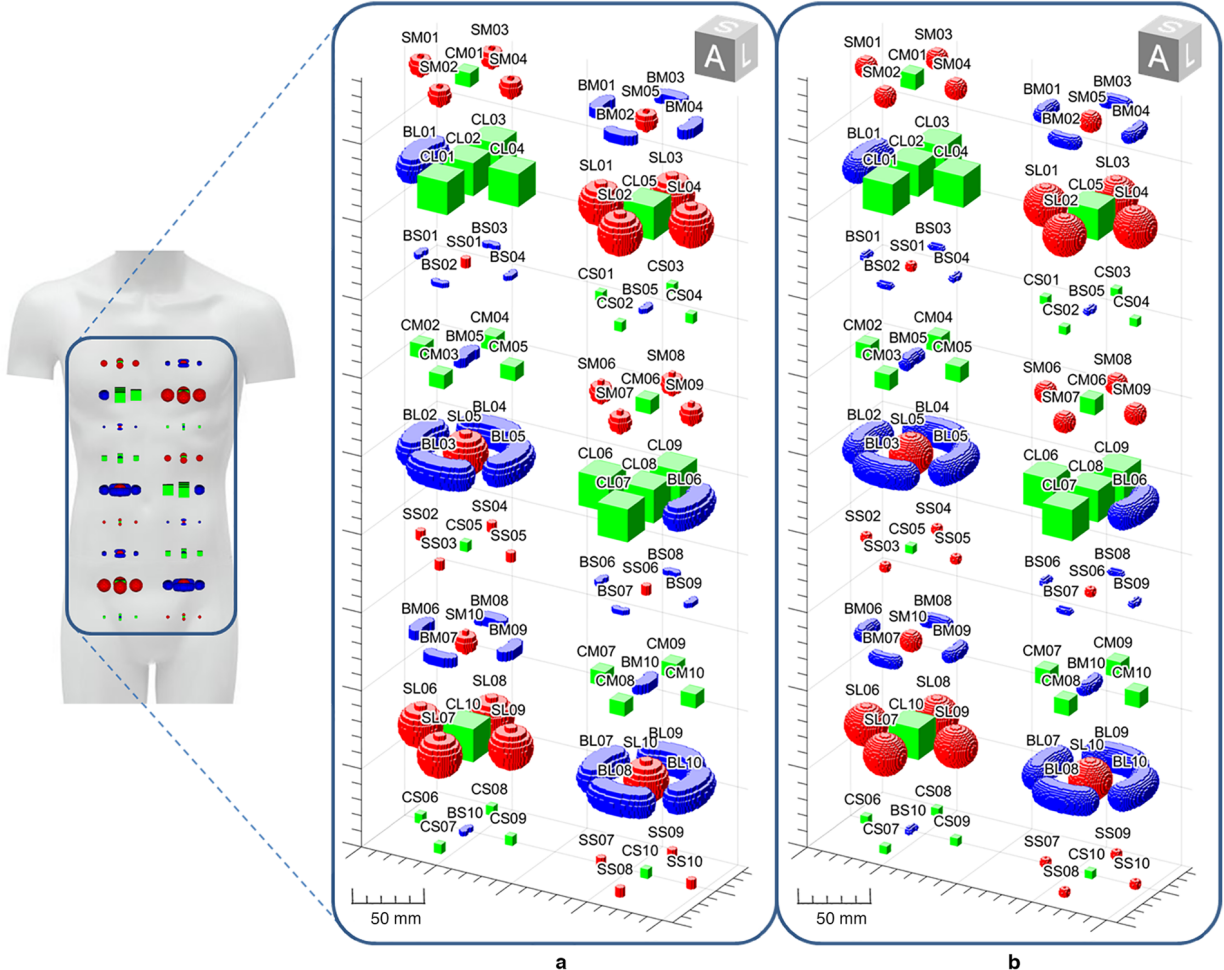
Spatial arrangement of the 90 ROIs with respect to the patient’s body. (**a**) ROI binary masks for the anisotropic phantom. (**b**) ROI binary masks for the isotropic phantom.

The 9 surface models were imported into MATLAB (the MathWorks, Natick, 2020a) and converted into binary masks. For this purpose, two three-dimensional point grids representing both the anisotropic and isotropic voxel centers were built and intersected with the nine surface meshes. Voxels of the grid whose center fell inside the mesh were set to 1, otherwise to 0. Afterwards, ten repetitions for each of the nine binary masks were positioned over the space of each CT image and were axially arranged to create 18 different groups of five ROIs each (see Fig. 3).

Eventually, only the texture of the underlying CT image within and around each ROI (with a margin of 6 mm) was kept, while the surrounding voxels were censored by setting their intensity to −1024 Hounsfield Unit (HU). The resulting censored images and the two binary masks constitute the two ImSURE phantoms.

**Fig. 4:**
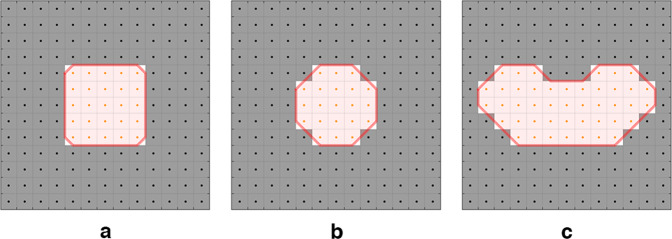
Definition of the contour set from the binary masks. Red lines indicate the generated contours for the central slice of the small cube (**a**), sphere (**b**) and bean (**c**) shapes.

The binary masks of the 90 ROIs were converted to sets of contour points to create the RTstruct file necessary for the DICOM format. For each slice of the binary masks, the external contour of the ROI was traced using a self-developed MATLAB code that drew the contour line between the center of the last voxel included in the binary mask and that of the first voxel outside the mask. Figure 4 shows the contour line for the central slices of three representative binary masks corresponding to the small ROI shapes.

**Fig. 5:**
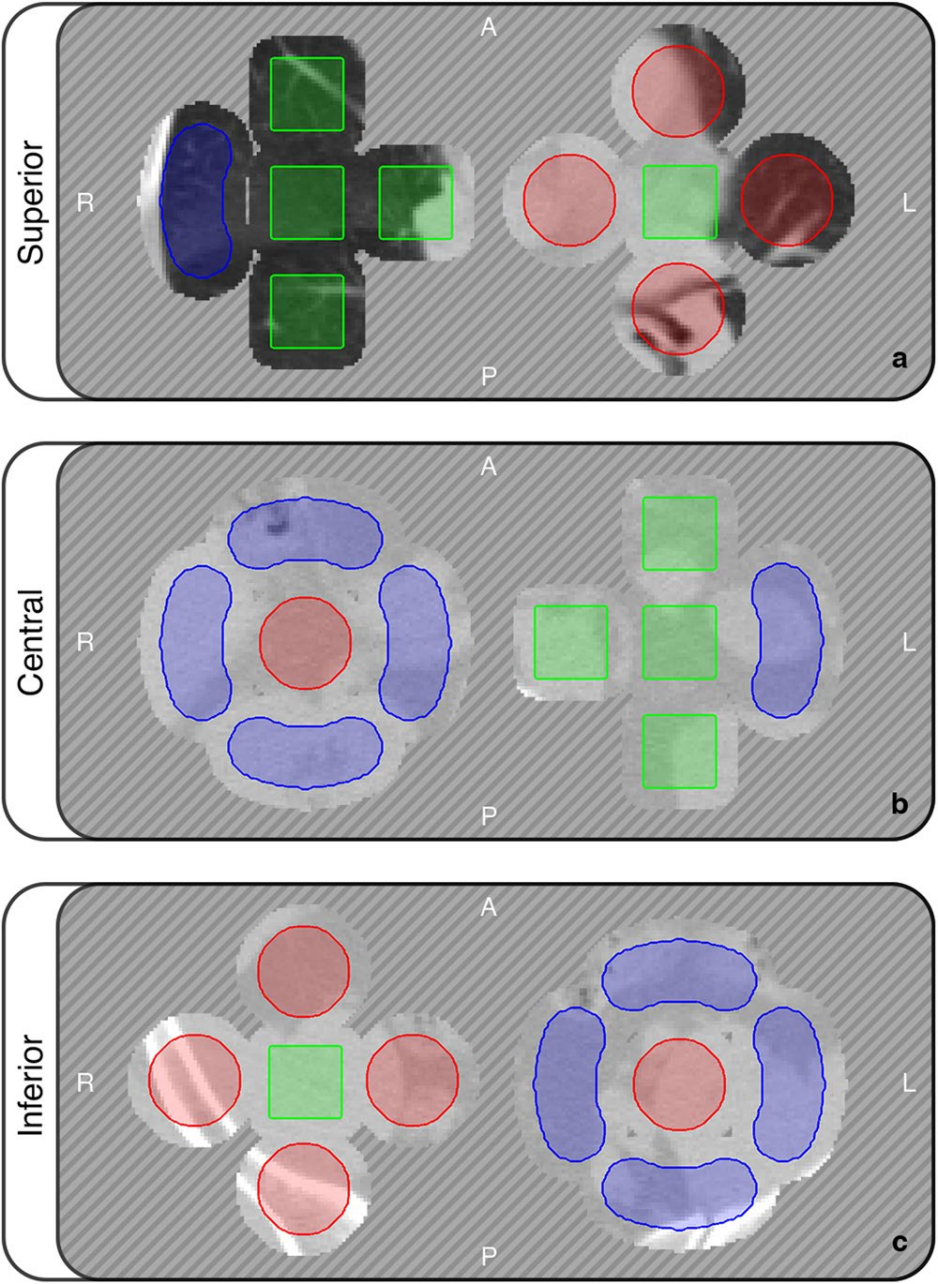
Three slices of the isotropic phantom containing the central portions of large ROIs. The segmentation is superimposed on the image textural content. Diagonal lines represent the censored CT data. A: anterior, P: posterior, R: right, L: left. Window: [−1000, 400] HU.

The final characteristics of each base morphology are summarised in Table 1, while three representative slices of the isotropic phantom are visible in Fig. 5.

**Table 1:** Characteristics of the 9 different ROI configurations defined for both the isotropic and the anisotropic phantom.

Feature family	Isotropic phantom	Anisotropic phantom
**Total Number of ROI**	90	90
**Voxel spacing [mm]**	1.00 × 1.00	0.977 × 0.977
**Slice Thickness [mm]**	1.00	3.00
**Voxel volume [mm3]**	1	2.86
**ROI Volumes [mm3]**		
*Bean - Small*	120	125.89
*Bean - Medium*	998	881.20
*Bean - Large*	7964	7896.43
*Sphere - Small*	123	120.16
*Sphere - Medium*	1020	975.61
*Sphere - Large*	8025	7990.85
*Cube - Small*	125	143.05
*Cube - Medium*	1000	858.31
*Cube - Large*	8000	8010.87

The volumes reported in the table correspond to the number of voxels in each ROI multiplied by voxel dimension.

#### DICOM export and format conversion

The censored CT images and their respective contour sets were saved in DICOM format using MATLAB 2020a. Eventually, 3D Slicer (version 4.10, http://www.slicer.org) software was used to convert the DICOM files to NIfTI and NRRD formats, so as to guarantee ease of use of our phantoms with different software tools. Depending on the file format, ROI unique identifiers (indicating shape, volume and instance number) were stored within the file header, in the mask file name or in a separate file.

### Design of feature-extraction workflow

Our phantoms were ad-hoc designed to test radiomic software in a heterogeneous scenario: the 90 ROIs with different morphological characteristics have been specifically thought for the identification of differences in feature values among radiomic programs due to ROI morphology (i.e. ROI shape and volume) that could not be investigated with previous datasets.

To complement the ImSURE anisotropic and isotropic phantoms, we designed two systematic workflows of feature extraction (with and without interpolation, respectively), which allow to investigate the impact of pre-processing configurations on the reproducibility of features across software programs.

Given a phantom, which may or may not require interpolation, the feature extraction procedure was designed to include all possible combinations of pre-processing steps, namely two intensity discretisation approaches (i.e. FBN or FBS) combined with all feature aggregation methods (i.e. 2D:avg, 2D:mrg, 2.5D:avg, 2.5D:mrg, 3D:avg, 3D:mrg, 2D, 2.5D, 3D). For every method, specific parameters were chosen among the most employed in the radiomic literature (e.g. for FBS we used a bin width of 25 HU). Parameter explanation and details can be found in the IBSI reference manual^2^.

From the 169 IBSI-standardised radiomic features, considering all the possible combinations of the extraction parameters, we obtained a total of 919 feature values for each ROI.

**Fig. 6:**
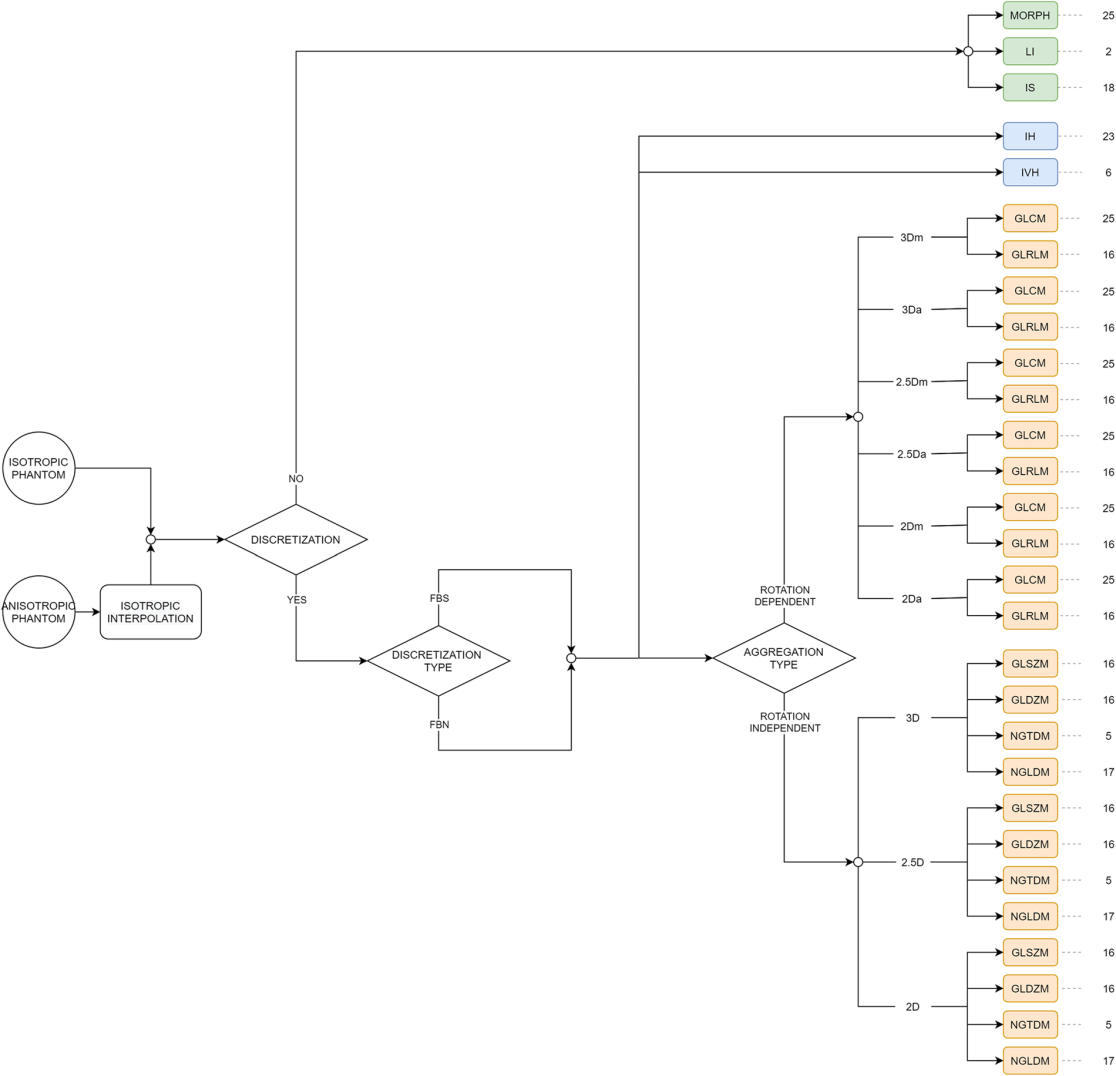
Scheme depicting the proposed feature extraction comprehending 919 features values. (FBN = fixed bin number; FBS = fixed bin size; MORPH = morphology; LI = local intensity; IS = intensity-based statistics; IH = intensity histogram; IVH = intensity-volume histogram; GLCM = grey level co-occurrence matrix; GLRLM = grey level run length matrix; GLSZM = grey level size zone matrix; GLDZM = grey level distance zone matrix; NGTDM = neighbourhood grey tone difference matrix; NGLDM = neighbouring grey level dependence matrix).

Table 2 synthesises the proposed extraction settings for the two phantoms, while Fig. 6 presents the scheme of the extracted features in better detail.

**Table 2:** Pre-processing settings used for the isotropic and anisotropic phantoms.

Pre-processing step	*Isotropic phantom*	*Anisotropic phantom*
**Trilinear Interpolation**		
*resampled voxel spacing [mm]*	none	1.00 × 1.00 × 1.00
**Re-segmentation**		
*range [HU]*	[−1000 400]	[−1000 400]
**Discretisation**		
*texture and IH*	FBS: 25 HU; FBN: 32 bins	FBS: 25 HU; FBN: 32 bins
*IVH*	FBS: 2.5 HU; FBN: 1000 bins	FBS: 2.5 HU; FBN: 1000 bins

(FBN = fixed bin number; FBS = fixed bin size; HU = Hounsfield Unit; IH = intensity histogram feature family; IVH = intensity-volume histogram feature family).

### Calculation of reference feature values

The radiomic tools that were selected to compute reference feature values were MIRP, S-IBEX, RaCaT, SERA and Pyradiomics. For each phantom, feature extraction was performed by tuning the parameters of the five radiomic software programs to match the aforementioned configurations.

#### Selected radiomic tools

We resorted to the latest version available of the five open-source software (i.e., MIRP v1.0.2, S-IBEX v2, RaCaT v1.18, SERA v2.1 and Pyradiomics v3.0.1) to compute the reference feature values for the two ImSURE phantoms. All of the selected tools were IBSI-participants^2^ (i.e., took part in the IBSI standardisation process) and/or independently declared their IBSI-compliance^9,10^. Nevertheless, they have different degrees of standardisation, for example, Pyradiomics declares in its documentation that it does not resort to a IBSI-compliant resampling method (https://pyradiomics.readthedocs.io/en/latest/index.html).

MIRP was the leading software used in the IBSI study; it is based on Python language and specific pre-processing parameters can be set by filling a configuration file. S-IBEX (v2) is the updated and IBSI-compliant version of IBEX^14^, which is currently in use in our institute and is developed in MATLAB. Thanks to a user-friendly graphical user interface, it is possible to easily specify the desired configuration settings. RaCaT is a radiomic calculator written in C++ as a standalone executable. Extraction parameters have to be set by filling a specific configuration file. SERA is a library developed for MATLAB and only works on medical images already imported into MATLAB, thus the importing step is left to the user and requires external functions, while parameters must be set within the SERA main code. Pyradiomics is presently one of the most common radiomic libraries and similarly to MIRP, it is written in Python. The specific parameters for feature extraction can be set both inside the code or with a dedicated configuration file.

For each software, the IBSI compliance, namely the matching percentages with the IBSI reference values calculated on the *IBSI digital* and *radiomic phantoms*, are reported in Table 3, which shows that, among the five considered tools, MIRP and S-IBEX achieved the highest number of matches with the IBSI benchmark values.

**Table 3:** Percentages of matching, partial matching, no matching, and missing feature values obtained for each software package on the *IBSI digital and radiomic phantoms*.

	IBSI digital Phantom (482 values)				IBSI radiomic phantom (1322 values)			
	Match [%]	Partial match [%]	No match [%]	Missing [%]	Match [%]	Partial match [%]	No match [%]	Missing [%]
MIRP	100	0	0	0	99.85	0.08	0.08	0
S-IBEX	100	0	0	0	99.85	0.08	0.08	0
RaCaT	94.40	0.41	4.56	0.62	89.71	1.06	8.09	1.13
SERA	95.23	0	4.36	0.41	84.95	0.76	13.54	0.76
Pyradiomics	52.28	0.21	0.21	47.30	22.39	13.84	17.10	46.67

#### ImSURE reference values

On the ImSURE phantoms, of the 919 feature values, only MIRP and S-IBEX could compute all features, while RaCaT, SERA and Pyradiomics were able to extract 916, 917 and 479 feature values, respectively.

For MIRP and S-IBEX, the 97.7% of the features resulted in an exact match to the third significant digit for the isotropic phantom (that did not require program-specific interpolation ahead of feature calculation) and the 98.4% for the anisotropic phantom (which required program-specific resampling). These two software programs behave similarly when compared to the other three programs, i.e., to RaCaT, SERA and Pyradiomics, both presenting a percentage of exact matches with them of ~85%, ~52% and ~85%, respectively for the ImSURE isotropic phantom, and of ~84%, 26.4% and 1.5% respectively, for the ImSURE anisotropic phantom.

## Data Records

The ImSURE phantoms generated in this work are available in the public “*ImSURE Phantoms*” repository on Figshare^15^. The repository contains:

I. The ImSURE isotropic and anisotropic phantoms. To ease their usage with different radiomic tools, phantoms are available in DICOM, NIfTI, NRRD and MATLAB formats.
II. A document reporting the parameter settings necessary to reproduce the proposed feature extraction on the ImSURE phantoms.
III. The Reference feature values that were computed from the ImSURE phantoms using the five software programs. Features are reported in .xlsx and .csv file formats and organised by pre-processing configuration, feature family and software.

## Technical Validation

The patient CT image used to create the two phantoms was collected at the *Veneto Institute of Oncology* (IOV-IRCCS, Padova, Italy), where quality assurance is performed as part of routine patient healthcare. The adequacy of image anonymisation has been validated by the Information Technology Department of IOV.

It is worth noting that, even though the 90 ROIs do not exactly follow the anatomy, they were placed on the image space in order to sample different textures, thus we are confident that the range of features extracted from the ROIs on our phantoms spans the range of possible features values derivable from clinical datasets. Therefore, we may infer that the analytical outcomes obtained on our phantom can be translated in a clinical setting.

## Data Availability

The MATLAB code used to design the two phantoms presented in this work is available in the Figshare “*Phantom-Creator*” repository^16^. The MATLAB version 2020a and the Image Processing Toolbox are required to run the code.
